# Association between C-reactive protein and risk of schizophrenia: An updated meta-analysis

**DOI:** 10.18632/oncotarget.17995

**Published:** 2017-05-18

**Authors:** Zhichao Wang, Ping Li, Dianyuan Chi, Tong Wu, Zubing Mei, Guangcheng Cui

**Affiliations:** ^1^ Academic Research Department, Qiqihar Medical University, Qiqihar, Heilongjiang Province, China; ^2^ Department of Psychiatry, Qiqihar Medical University, Qiqihar, Heilongjiang Province, China; ^3^ Department of Anorectal Surgery, Shuguang Hospital, Shanghai University of Traditional Chinese Medicine, Shanghai, China

**Keywords:** C-reactive protein, schizophrenia, risk, meta-analysis

## Abstract

C-reactive protein (CRP) has been indicated to be associated with the pathogenesis of schizophrenia (SZ) and other psychiatric disorders. The aim of this study is to investigate whether peripheral blood CRP levels are associated with the risk of SZ. We searched literature from databases of Pubmed, Embase and the Cochrane Library from inception to November 1, 2016 for studies that reported serum or plasma CRP levels in patients with SZ and non-SZ controls. At least two reviewers decided on eligibility and extracted data from included studies. Random effects meta-analyses were performed using standardized mean difference (SMD) as the effect estimate of the differences in CRP levels between subjects with SZ and healthy controls. We identified 18 studies representing 1963 patients with SZ and 3683 non-SZ controls. Compared with non-schizophrenics, blood CRP levels were moderately increased in people with SZ (SMD 0.53, 95% CI 0.30 to 0.76) irrespective of study region, sample size of included studies, patient mean age, age of SZ onset and patient body mass index. Publication bias was not detected through Egger's linear regression test (*P* = 0.292). We noticed that patients in Asia or Africa (*n =* 6, SMD 0.73, 95% CI 0.26 to 1.21) and whose age less than 30 years (*n =* 5, SMD 0.76, 95% CI 0.07 to 1.58) had substantially higher CRP levels. Our study provides evidence that higher CRP levels are associated with increased risk of SZ, especially for young adult patients less than 30 years. Further large-scale studies are strongly warranted to further confirm this association.

## INTRODUCTION

Immune system dysfunctions and inflammatory processes have been implicated in the pathogenesis of schizophrenia (SZ) [1–5]. A vast observational studies, mainly case-control studies provide increasing evidence that patients with SZ have elevated levels of some inflammatory biomarkers, such as cytokines in plasma or serum [1, 4]. Furthermore, evidence from epidemiologic studies implied that some autoimmune diseases contributed to the development of SZ [6, 7]. As an acute-phase protein, C-reactive protein (CRP) is produced by some inflammatory stimula and is mainly induced by some pro-inflammatory cytokines [8]. Increased CRP levels have been reported in numerous observational studies in psychosis including SZ and in bipolar disorder [9]. Metcalf et al. reported that individuals with high (> 3 mg/L) compared with low (< 1 mg/L) CRP levels at baseline were more likely to develop SZ (adjusted odd ratio 4.25, 95% CI, 1.30 to 13.93) [55]. Zhang et al. found that high sensitivity CRP/ interleukin-10 was a potential peripheral biomarker of SZ [56].

It has also been indicated that the increase in serum or plasma CRP levels contributes the risk of a first episode of SZ [9]. Inoshita et al. conducted a mendelian randomisation study and found a causal relationship between increased CRP levels and the development of SZ [10]. However, several other studies reported no association between CRP levels and risk of SZ [11–13]. A meta-analysis by Fernandes et al published in 2015 of CRP levels in patients with SZ found that CRP was increased in SZ but was not altered by antipsychotics [14]. However, owing to the mixed studies combined for both case-control and cross-sectional studies in meta-analysis, we would like to update the evidence for only case-control studies published in English peer review journals.

## RESULTS

### Description of the included studies

**Figure 1:**
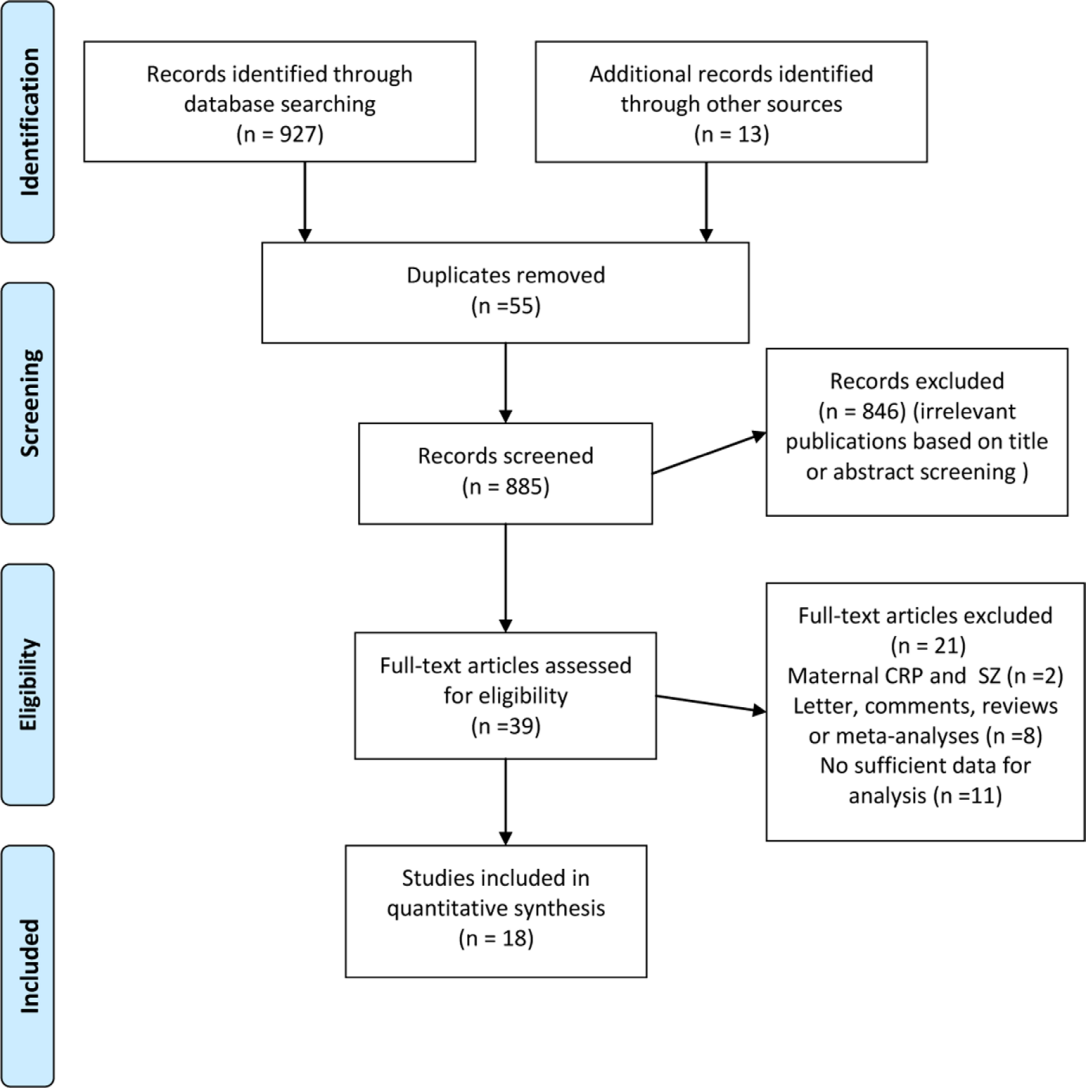
Preferred reporting items for systematic reviews and meta-analyses flow diagram depicting overview of study-selection process for studies reporting on C-reactive protein and risk of schizophrenia

The literature search yielded 927 citations. After excluding the duplicate, 39 appeared relevant and were retrieved for full-text review afer reading the titles or abstracts (Figure 1). In summary, 18 studies recruiting 1963 patients with SZ and 3683 non-SZ controls were enrolled in this meta-analysis [10–13, 15–28]. Detailed characteristics of individual studies are presented in Table 1. The sample size of included studies ranged from 60 to 1783 participants. Eleven studies were conducted in European countries, six in Asian or African countries and one in USA. Three studies recruted participants from inpatient unit, while another three from outpatient unit, eight from both units and four from population-based samples. Seven studies enrolled patients with age of SZ onset less than 30 years and two more than 30 years. Five studies investigated participants with body mass index less than 25kg/m^2^ and 11 studies with the range from 25 to 30 kg/m^2^. Most of the studies (17/18) applied high-sensitivity CRP assay except for one [13]. Twelve of the 18 included studies were of high quality and the remaining six studies were assessed as medium quality.

**Table 1:** Characteristics of the included studies in the meta-analysis investigating the association between C-reactive protein and risk of schizophrenia

Study	Year	Country	Subjects	Sex Male/Female	Setting	Meanage (years)	Meanage of onset (years)	Mean BMI	Adjusted variables for controls	Current Smokers (%)	CRP assay type	Comorbidities	Psychiatric drugs
Sarandol et al.,	2007	Turkey	Chronic SZ Control	18/22 17/18	Inpatient and outpatient unit	34.9 33.5	NA	23.7 24.6	Age, BMI, smoking status	NA	Plasma, high sensitive immunonephrelometry assay	Exclusions: DM and use of anti-inflammatory or, HAS, CVD, infections immunossupressants	Drug-naïve or free
Carrizo et al.	2008	Venezuela	Chronic SZ Control	48/40	Inpatient and outpatient unit	43.2 54.79	NA	27.5 28.6	No	NA	Serum, high sensitive enzime immunoassay	Exclusions: DM , HAS, CVD, infections and use of anti-inflammatory or immunossupressants	Typical and Atypical antipsychotics
Akanji et al., 2009	2009	Kuwait	Chronic SZ Control	141/62	Inpatient unit	141/62 165/0	NA	39.80 39.50	Age, race, socioeconomic status	NA	Serum, high sensitive Chemiliminescent immunometric assay	Exclusions: DM, HAS, CVD, infections and use of anti-inflammatory or immunossupressants	Typical antipsychotics
Fernandes-Egea et al., 2009	2009	Spain	SZ in FEP Control	35/15 35/15	Populational	29.4 28.8	29	22.9 23.9	Age, gender, BMI,number of cigarettes/day, catchment area	NA	Serum, not high sensitive immuno assay	DM (*n* = 9). Exclusions: other relevant clinical pathologies	
Fawzi et al., 2011	2011	Egypt	Chronic SZ Control	92/0 200/0	Outpatient unit	28.4 28.8	NA	27 26.4	Age, gender, BMI, lifestile	147 132	Plasma, high sensitive latex assay	Exclusions: DM , HAS, CVD, infectionsand use of anti-inflammatory orimmunos- supressants	Drug-naïve or free Drug-naïve
Hope et al., 2011	2011	Norway	Chronic SZ Control	89/64 105/134	Populational	36.2 36		26 24.4	Age, BMI, catchment area	86 NA	Plasma, high sensitive enzime immunoassay	DM (*n* = 4) and CVD (*n* = 11). Exclusions:other relevant clinical pathologies	Typical and Atypical antipsychotics
Suvisaari et al., 2011	2011	Finland	Chronic SZ Control	39/63 39/63	Populational	55.7 55.78	NA	28.6 26.4	Age, gender, BMI	37 27	Plasma, high sensitive immunotur- bidometric test	DM (*n* = 11). Exclusions: other relevant clinical pathologies, infections	Typical and Atypical antipsychotics
Hepgul et al. 2012	2012	England	SZ in FEP Control	NA	Inpatient and outpatient unit	33.5 33.1	NA	25.6 25.1	No	NA	Serum, high sensitive enzime immunoassay	Exclusions: DM , HAS, CVD, infectionsand use of anti-inflammatory or immunossupressants	Drug-free
Dickerson et al.,2013	2013	United States	Chronic SZ Control	177/118 84/144	Inpatient and outpatient unit	39 32.2	20.3	30.3 27.8	No	183 36	Serum, high sensitive enzime immunoassay	DM (*n* = 17). Exclusions: other relevant clinical pathologies	Typical and Atypical antipsychotics
Joshi et al., 2013	2013	India	Chronic SZ Control	29/16 21/20	Inpatient and outpatient unit	38.5 35.8	23.90	27.3 26.5	Age, BMI, catchment	NA	Plasma, high sensitive enzime immunoassay	Exclusions: DM , HAS, CVD, infectionsand use of anti-inflammatory or immunossupressants	Typical antipsychotics
Kuo et al., 2013	2013	Taiwan	Chronic SZ Control	19/14 12/18	Outpatient unit	37.8 38.8	33.90	29.5 28.3	Age, BMI	NA	Plasma, high sensitive latex assay	Exclusions: DM , HAS, CVD, infections and use of anti-inflammatory or immunossupressants	Typical and Atypical antipsychotics
Lin et al., 2013	2013	Taiwan	Chronic SZ Control	16/20 16/20	Inpatient and outpatient unit	35.7 37.3	23.79	24.6 22.9	Age, gender, BMI,	NA	Serum, high sensitive immunonephrelometry assay	Exclusions: DM , HAS, CVD, infectionsand use of anti-inflammatory or immunossupressants	Atypical antipsychotics
Vuksan-Cusa et al., 2013	2013	Croatia	Chronic SZ Control	NA	Inpatient unit	26.9 24.9	15.7	26.9 24.9	Age, gender, BMI,	NA	Plasma, high sensitive immunoturbidometric test	Exclusions: DM , HAS, CVD, infectionsand use of anti-inflammatory or immunossupressants	Atypical antipsychotics
Berardis et al., 2014	2014	Italy	SZ in FEP Control	13/17 13/17	Inpatient unit	25.9 25.5	24.90	22.1 23.5	Age, gender	NA	Plasma, high sensitive immunonephrelometry assay	Exclusions: DM , HAS, CVD, infections and use of anti-inflammatory or immunossupressants	Drug-naïve
Frydecka et al., 2014	2014	Poland	Chronic SZ Control	69/82 103/91	Inpatient and outpatient unit	37.8 39.2	25.20	27.11 26.9	Age, gender, BMI	NA	Serum, high sensitive enzime immunoassay	Exclusions: DM, HAS, CVD, infections and use of anti-inflammatory or immunossupressants	NA
Klemettila et al., 2014	2014	Finland	Chronic SZ Control	105/85 403/500	Inpatient and outpatient unit	42.9 46	NA	29.92 26.5	No	101 NA	Plasma, high sensitive enzime immunoassay	Exclusions: DM, HAS, CVD, infectionsand use of anti-inflammatory or immunossupressants	Clozapine
Stojanovic et al., 2014	2014	Spain	SZ in FEP Control	48/29 12/13	Outpatient unit	24.3 27.3	NA	24.4 21.6	No	53	Plasma, high sensitive immunoturbidometric test	NA	Typical and Atypical antipsychotics
Inoshita et al., 2016	2016	Japan	Chronic SZ Control	241/177 422/943	Populational	62.5 62.6	NA	NA	Age, gender	NA	Serum, high sensitive enzime immunoassay	Exclusions: CRP concentration either below 0.02 mg/dl or above 10 mg/dl	Typical and Atypical antipsychotics

Abbreviations: BMI, body-mass index; CRP, C-reactive protein; CVD, cardiovascular diseases; DM, Diabetes Mellitus; HAS, hypertension; HDRS, Hamilton Depression Rating Scale; NA, not available; YMRS, Young Mania rating Scale.

### Meta-analysis of Association between CRP and Risk of Schizophrenia

**Figure 2:**
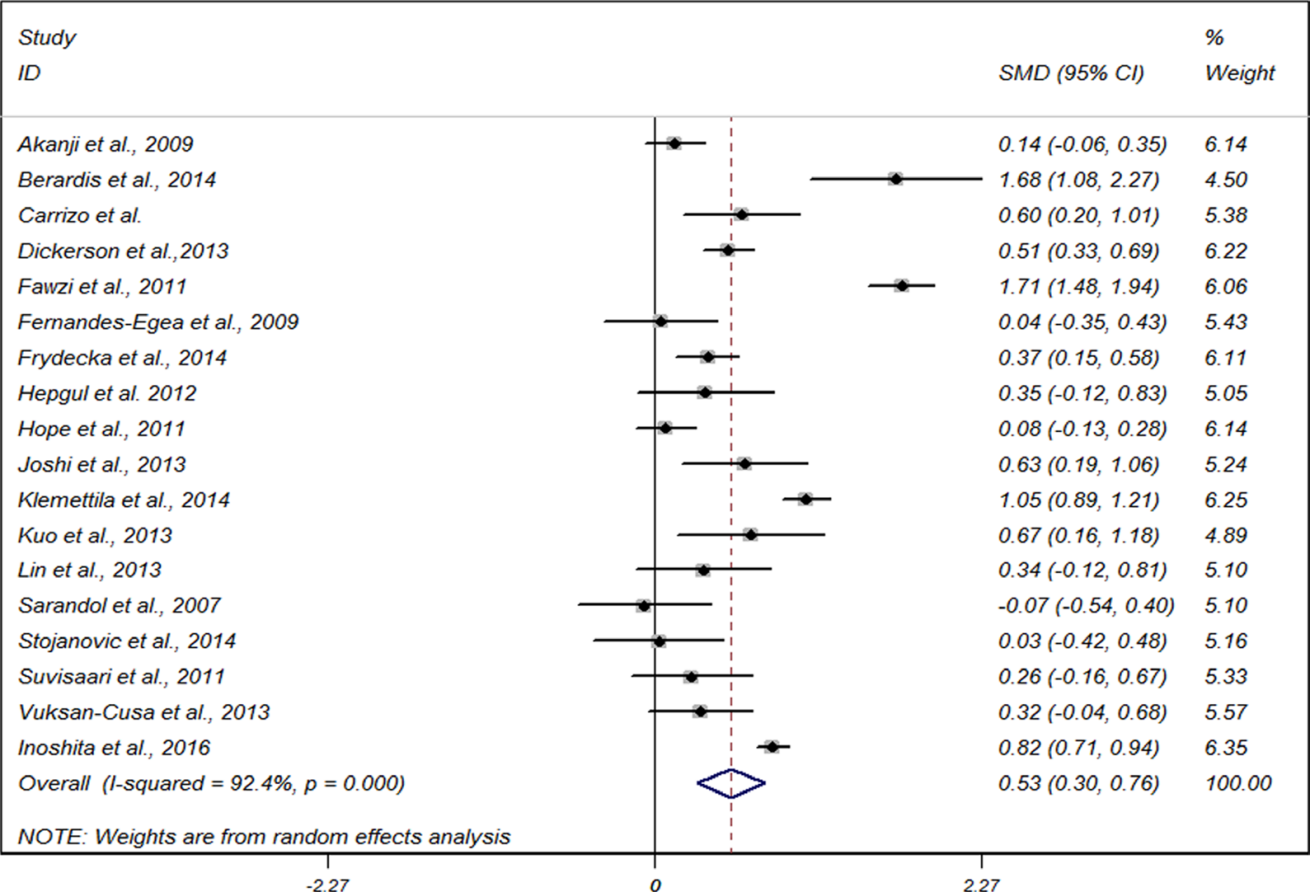
Forest plot for meta-analysis of the association between C-reactive protein and risk of schizophrenia with the use of a random-effects model SMD, standardized mean difference; CI, confidence interval.

Meta-analysis based on 18 studies, showed that compared with non-schizophrenics, CRP levels were moderately increased in people with SZ (standardized mean difference (SMD) 0.53, 95% CI 0.30 to 0.76), with significant heterogeneity among studies (I^2^ = 92.5%, *P* < 0.001) (Figure 2). We then conducted subgroup analyses stratified by study region, sample size, clinical setting, mean age, age of SZ onset, body mass index, adjusted controls and high-sensitivity CRP assay used or not. The findings did not largely alter compared with that of the main analysis for most of the subgroups (Table 2). A bordine estimates were seen for studies for clinical setting of inpatient unit (SMD 0.66, 95% CI–0.05 to 1.38) and outpatient unit (SMD 0.82, 95% CI–0.05 to 1.38).

**Table 2:** Main results of subgroup analyses to explore sources of heterogeneity based on some investigated variables for association between C-reactive protein levels and risk of schizophrenia

Variables	Heterogeneity (I^2^ %; *P*_het_)	SMD 95% CI	*P*_interaction_
Total	18 (92.5; < 0.001)	0.53 (0.30 to 0.76)	NA
Study region			< 0.001
USA	1 (–, –)	0.51 (0.33 to 0.69)	
Europe	11 (89.6, < 0.001)	0.42 (0.12 to 0.72)	
Asia/Africa	6 (95.3, < 0.001)	0.73 (0.26 to 1.21)	
Sample size			0.075
≥ 100	10 (95.5, < 0.001)	0.52 (0.21 to 0.83)	
< 100	6 (77.7, < 0.001)	0.63 (0.22 to 1.03)	
Clinical setting			< 0.001
Inpatient unit	3 (91.6%, < 0.001)	0.66 (–0.05 to 1.38)	
Outpatient unit	3 (92.5%, < 0.001)	0.82 (–0.28 to 1.93)	
Both units	8 (84.3, < 0.001)	0.50 (0.24 to 0.76)	
Population-based	4 (95.9, < 0.001)	0.31 (0.17 to 0.80)	
Mean age			< 0.001
< 30 years	5 (95.9, < 0.001)	0.76 (0.07 to 1.58)	
30-50 years	11 (87.7, < 0.001)	0.43 (0.19 to 0.67)	
≥ 50 years	2 (84,6, < 0.001)	0.58 (0.03 to 1.12)	
Age of onset			< 0.001
≤ 30 years	7 (74.6, 0.001)	0.50 (0.25 to 0.75)	
> 30 years	2 (0, 0.591)	0.81 (0.70 to 0.93)	
Body mass index			< 0.001
≤ 25 kg/m^2^	5 (85.1, < 0.001)	0.38 (0.16 to 0.92)	
25–30 kg/m^2^	11 (93.5, < 0.001)	0.60 (0.29 to 0.92)	
Adjusted controls			0.077
Yes	13 (93.7, < 0.001)	0.53 (0.23 to 0.84)	
No	5 (87.8, < 0.001)	0.54 (0.19 to 0.90)	
High-sensitivity CRP assay			0.003
Yes	17 (92.6, < 0.001)	0.56 (0.33 to 0.80)	
No	1 (–, –)	0.04 (–0.35 to 0.43)	

Abbreviations: CI, confidence interval; CRP, C-reactive protein; het, heterogeneity; NA, not available; SMD, standardized mean difference.

### Study region

Subgroup analysis stratified by study region showed that serum and plasma CRP levels were increased moderately in studies conducted in European countries (*n* =11, pooled SMD 0.42, 95% CI 0.12 to 0.72) and significantly in Asian/African countries (*n* = 6, pooled SMD 0.73, 95% CI 0.26 to 1.21). We found statistically significant difference for inter-study heterogeneity (*P* < 0.001).

### Clinical setting

Meta-analysis stratified by clinical setting demonstated that serum and plasma CRP levels were increased moderately in studies performed in combination with inpatient and outpatient unit (*n* = 8, pooled SMD 0.50, 95% CI 0.24 to 0.76) and slightly in population-based samples (*n* = 4, pooled SMD 0.31, 95% CI 0.17 to 0.80). We found statistically significant difference for inter-study heterogeneity (*P* < 0.001).

### Sample size

Subgroup analysis stratified by sample size indicated that serum and plasma CRP levels were increased moderately in studies with large sample size (≥ 100) (*n =* 10, pooled SMD 0.52, 95% CI 0.21 to 0.83); similar results were also obtained for studies with small sample size (< 100) (*n =* 6, pooled SMD 0.63, 95% CI (0.22 to 1.03).We found no statistically significant difference for inter-study heterogeneity (*P* = 0.075).

### Age

Subgroup analysis stratified by age indicated that serum and plasma CRP levels were increased significantly in studies with participants’age <30 years (*n =* 5, pooled SMD 0.76, 95% CI 0.07 to 1.58); but moderately in studies with participants’age ranging from 30 to 50 years (*n =* 11, pooled SMD 0.43, 95% CI 0.19 to 0.67); similar results were also obtained for studies with participants’age ≥ 50 years (*n =* 2, pooled SMD 0.58, 95% CI 0.03 to 1.12). We found statistically significant difference for inter-study heterogeneity (*P* < 0.001).

### Age of SZ onset

Subgroup analysis stratified by age of SZ onset showed that serum and plasma CRP levels were increased moderately in studies with participants’ age of SZ onset ≤ 30 years (*n =* 7, pooled SMD 0.50, 95% CI 0.25 to 0.75); but significantly in studies with participants’age ranging from 30 to 50 years (*n =* 11, pooled SMD 0.43, 95% CI 0.19 to 0.67); similar results were also obtained for studies with participants’ age of SZ onset > 30 years (*n =* 2, pooled SMD 0.81, 95% CI 0.70 to 0.93). We found statistically significant difference for inter-study heterogeneity (*P* < 0.001).

### Adjusted variables for controls

Subgroup analysis stratified by adjusted variables for controls showed that serum and plasma CRP levels were increased moderately in studies with (*n =* 13, pooled SMD 0.53, 95% CI 0.23 to 0.84) and without adjusted variables for controls (*n =* 5, pooled SMD 0.54, 95% CI 0.19 to 0.90). We found no statistically significant difference for inter-study heterogeneity (*P <* 0.077).

### Sensitivity analysis and publication bias

**Figure 3:**
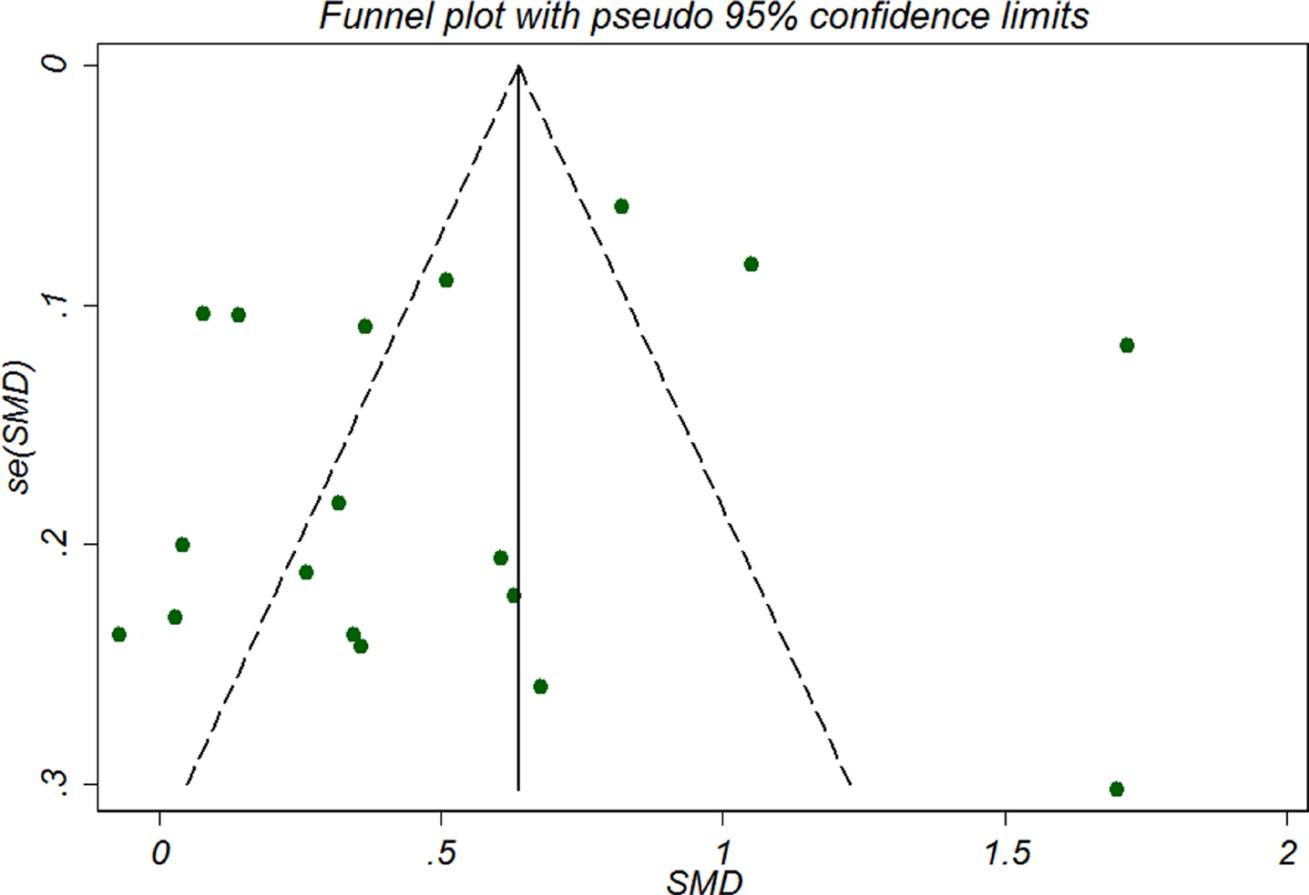
Funnel plot of publication bias in the selection of studies for assessing the relationship between C-reactive protein and risk of schizophrenia SMD, standardized mean difference.

Sensitivity analysis showed that serum and plasma CRP levels were increased moderately in studies involved only high-sensitivity CRP assay (*n =* 17, pooled SMD 0.56, 95% CI 0.33 to 0.80). No significant funnel plot asymmetry was detected as was shown in Figure 3, indicating no publication bias, which was further confirmed by Begg’s rank correlation test (*P =* 0.544) and Egger’s regression test (*P =* 0.292). Duval and Tweedie’s trim and fill method indicated that no missing study was inputed and the adjusted SMD was the same as the primary one, confirming the robustness of the analysis.

## DISCUSSION

In this meta-analysis based on 18 case-control studies, we found that serum and plasma CRP levels were moderately increased in patients with SZ, irrespective of study region, sample size of included studies, patient mean age, age of SZ onset and patient body mass index. We noticed that patients in Asia or Africa and whose age less than 30 years were more substantially increase in CRP levels.

The potential biological mechanism for the associations between some inflammatary factors and SZ has not been fully understood. Hsuchou et al. found that high peripheral levels of CRP could increase the permeability of the blood–brain barrier through the adjustment of the function of tight junctions, which contributed to the increase in some pro-inflammatory cytokines, such as CRP to enter the central nervous system [29].

The high levels of CRP in central nervous system has been reported to play an important part in some psychiatric dysfunctions, such as SZ [30, 31]. Experimental study showed that CRP affected viability of microglia and astrocytes, accelerating cell gliosis [32], thus leading to the release of some pro-inflammatroy factors, such as IL-6 and transforming growth factor-β. In addition, reports also showed that elevated CRP levels could influence the microcirculatory system in the blood–brain barrier, thus affecting neurotransmitter synthesis and neurotransmission [33–38]. Moreover, elevated CRP levels were reported to be associated with the severity of clinical symptoms, cognitive and sensory impairments in SZ [39–42]. The rationale that plasma CRP levels were increased significantly in studies with participants’age less than 30 years probably lies in that in the early stages of SZ, a particularly large number of inflammatory substances will be secreted, such as blood CRP and interleukin-10, which are very likely to be related to the development of SZ [57].

### Strength of the study

This updated literature search identified two systematic review and meta-analyses on the same topic [14, 43], but both of them focused on studies with mixed study design. Though solely one newly published study has been added to this analysis [10], this is a quite large sample size case-control study (418 cases/1365 controls) with greater statistical power. The findings of our studies are consistent with those two meta-analyses from mixed cross-sectional studies and case–control studies (60). Miller et al. [43] found that CRP levels were slightly to moderately increased in people with SZ (SMD 0.45, 95% CI 0.34 to 0.55) by pooling 8 cross-sectional studies, and *Fernandes* et al. [14] concluded that CRP levels were moderately to significantly increased in persons with SZ (SMD 0.66, 95% CI 0.43 to 0.88). Strength of our meta-analysis lies in the following three aspects. Firstly, most of the included studies in the previous meta-analyses were cross-sectional in study design. An known limitation of a cross-sectional study is that exposure and outcome are generally evaluated simutaneously and we therefore cannot draw conclusions on the casual-relationship between exposure and outcome. Hence, the underlying hypothesis obtained from the previous meta-analyses is that the prevalance of SZ is an immediate outcome following exposure of elevated CRP levels. The investigation of the real effects caused by continuous long-term exposure may require a different study design, such as our case-control study or cohort study [44]. Secondly, heterogeneity was noted among the included studies but we tried to account for this variation by conducting sensitivity analysis. The asymmetry of funnel plot indicated the overestimation of the effect size due to the lack of negative studies with small sample size. However, Begg’s rank correlation test and Egger’s regression test suggested little evidence of publication bias. Moreover, when applying the trim and fill method, no additional hypothised negative studies with small sample size were inputed, suggesting the robustness of the meta-analysis. Thirdly, it is the first study to suggest that younger patients with SZ whose age less than 30 years were more substantially increase in CRP levels. Though this potential reason is unclear, it has been suggested to be related with a greater reactivity of the immune system in younger patients [45]. However, further large well designed studies are warrented to validate this association.

Some inherent limitations do exist in our study. We report an increase in serum and plasma CRP levels in patients with SZ by pooling the eligible studies over the last decade. In our meta-analysis, we found the pooled SMD was 0.53 (95% CI 0.30 to 0.76) for the association between serum and plasma CRP levels and risk of SZ from the 18 studies included. The study quality of evidence obtained from NOS score was moderate and derived from case-control studies. However, still some other confounders remained due to factors that could not be accounted for such as physical activity, waist circumference, smoking habit, vitamin D intake, and subclinical infections. Most studies included in our analysis did not provide such data, which could explain the heterogeneity noted in the pooled analysis. Although subgroup analyses have been performed for some of the existing confounders, the investigated factors could only explain some of the heterogeneity. Moreover, many of the other analyses, such as dose-response relationship could not be conducted due to the lack of detailed information from original reports. Furthermore, the possible sources of heterogeneity was explored through subgroup analyses and sensitivity analyses. However, the results of these two methods did not allow us to attribute heterogeneity to any single study as the sole source of the high heterogeneity we obtained in most analyses. Secondly, this updated meta-analysis on serum and plasma CRP levels in patients with SZ compared with non-SZ controls provided us with summary estimates originating from one type of observational study (case-control study). Therefore, no definite causal conclusion can be drawn from this meta-analysis. High serum and plasma CRP level is only one of the inflammation-related factors known to be associated with the risk of SZ. Other factors such as smoking habit and subclinical infections could also be additional related factors. Thirdly, like any meta-analysis, this one is also dependent on the quality of the included studies, and our findings need to be further confirmed by studies specifically designed to demonstrate the unclear points we raised. Finally, the restriction of our meta-analysis to English language studies may have led to language bias with potentially relevant studies published in other languages being missed.

In summary, our meta-analyses provide evidence that higher CRP levels are associated with increased risk of SZ, especially for young adult patients less than 30 years. Future studies should be focused on whether changes in CRP levels have a causal relationship with the development of SZ.

## MATERIALS AND METHODS

### Literature search and study selection

The Preferred reporting items for systematic reviews and meta-analyses (PRISMA) statement was used when we performed this systematic literature review [46]. We systematically searched databases of Pubmed, EMBASE and the Cochrane Library from inception till November 1, 2016 for relevant studies, using the predefined search strategies (Supplementary search strategy 1–3). The following MeSH terms combined with free text words were used:schizophrenia /schizophreni* /schizoid /psychosis /schizophreniform/schizoaffective/psychotic disorders and C-reactive protein /CRP /hsCRP /hs-CRP. Additional related journal articles were identified by manual scanning of reference lists of articles, and the journals selected were JAMA Psychiatry, American Journal of Psychiatry, Molecular Psychiatry, British Journal of Psychiatry and European Psychiatry. We only included studies in English peer-review journals.

Studies were considered eligible in the meta-analysis according to the following criteria: 1) adult patients diagnosed with SZ based on Diagnostic and Statistical Manual for Mental Disorder-Fourth Edition-Text Revised (DSM–IV–TR) were enrolled in the study; 2) case-control studies with the non-SZ comparisons measuring serum or plasma CRP levels. We excluded studies with duplicate data or studies that reported CRP levels using dichotomous data (ie, positive or negative), or studies without a control group. In addition, we did not include unpublished literature due to the insufficient data obtained from these studies.

### Data extraction and bias assessment

Two authors (Z.W. and P.L.) independently searched, identified the related studies, extracted data and evaluated the study quality. When discrepancies occured, a third author (G.C.) made the definitive decision for study eligibility and data extraction. We extracted the following data including first author, publication year , research country, study subjects, clinical setting, sex, age and BMI of the study subjects, age of SZ onset, adjusted variables for controls, percent of the current smokers, CRP assay type, comorbidities and psychiatric drugs used. If necessary, we consulted the original data from the authors of the studies to collect missing information. The Newcastle–Ottawa Scales (NOS) was used to assess quality of the included studies [47], which applied three domains for assessment and allocated a total score of nine points. Quality categories were determined by the NOS score of each study. We defined that the score of high quality with 7 or more, medium quality from 4 to 6 and low quality less than 4.

### Statistical analysis

For continuous outcomes reported on different measurement methods for CRP levels, we applied standardised mean difference (SMD) estimates of the differences in CRP levels between patients with SZ and non-SZ controls as the effect size. The value of SMD being 0.2 was set to indicate a slight effect, meaning a small difference in CRP levels between patients with SZ and non-SZ controls, 0.5 a moderate effect, and 0.8 a significant effect [48]. The heterogeneity among the included studies was investigated using I^2^ and *Q* test statistics, with an I^2^ more than 50% or a *P* value less than 0.1 from Cochran’s Q-test indicating significant heterogeneity [49]. Summary estimates in the present meta-analysis were pooled using a random-effects model due to the predictable significant inter-study difference in the enrolled population, study design, or the treatment strategy [50]. We used funnel plots to visually inspect publication bias, along with Begg’s rank correlation test [51] and Egger’s regression test [52]. We also estimated the number of potential missing studies from a meta-analysis using the trim and fill method [53]. Some planned subgroup analyses were performed to explore possible causes of heterogeneity and between-subgroup interactions were calculated using the chi-squared significance test [54]. All meta-analyses were conducted using the software STATA version 12.0 (StataCorp LP, College Station, TX).

## SUPPLEMENTARY MATERIALS TABLES


