# Supplementary material for: Association between C-reactive protein and risk of schizophrenia: An updated meta-analysis
Source: Oncotarget. 2017 May 18;8(43):75445–54. doi: 10.18632/oncotarget.17995 (PMC5650435; doi:10.18632/oncotarget.17995)
Supplement: Supplementary file 1 [file oncotarget-08-75445-s001.pdf]

Association between C-reactive protein and risk of schizophrenia: an updated meta-analysis

SUPPLEMENTARY MATERIALS

Supplementary search strategy 1: Search strategy for pubmed

1. “ Schizophrenia”[Mesh]
2. “ Psychotic Disorders”[Mesh]
3. 1 OR 2
4. (schizophreni* OR schizoid OR psychosis OR schizophreniform OR schizoaffective) [Title/Abstract]
5. 3 OR 4
6. “ C-Reactive Protein “[Mesh]
7. (CRP OR C-reactive protein OR hsCRP OR hs-CRP) [Title/Abstract]
8. 6 OR 7
9. 5 AND 8

Supplementary search strategy 2: Search strategy for embase

1. ‘schizophrenia’/exp
2. ‘psychosis’/exp
3. 1 OR 2
4. (schizophreni* OR schizoid OR psychosis OR schizophreniform OR schizoaffective):ab,ti
5. 3 OR 4
6. ‘c reactive protein’/exp
7. (CRP OR C-reactive protein OR hsCRP OR hs-CRP):ab,ti
8. 6 OR 7
9. 5 AND 8

Supplementary search strategy 3: Search strategy for the cochrane library

1. MeSH descriptor: [Schizophrenia] explode all trees
2. MeSH descriptor: [Psychotic Disorders] explode all trees
3. 1 OR 2
4. schizophreni* or schizoid or psychosis or schizophreniform or schizoaffective:ti,ab,kw (Word variations have been searched)
5. 3 OR 4
6. MeSH descriptor: [C-Reactive Protein] explode all trees
7. CRP or C-reactive protein or hsCRP or hs-CRP:ti,ab,kw (Word variations have been searched)
8. 6 OR 7
9. 5 AND 8
